# Ultrasound insonation angle and scanning imaging modes for imaging dental implant structures: A benchtop study

**DOI:** 10.1371/journal.pone.0270392

**Published:** 2022-11-29

**Authors:** Oliver D. Kripfgans, Nikhila Devi Goli, Jad Majzoub, Rafael Amorim Cavalcanti De Siqueira, Fabiana Soki, Hsun-Liang Chan

**Affiliations:** 1 Department of Radiology, Michigan Medicine, University of Michigan, Ann Arbor, MI, United States of America; 2 Department of Periodontics and Oral, Medicine, School of Dentistry, University of Michigan, Ann Arbor, MI, United States of America; 3 Department of Periodontics, The Virginia Commonwealth University, Richmond, Virginia, United States of America; International Medical University, MALAYSIA

## Abstract

**Introduction:**

High frequency ultrasound has shown as a promising imaging modality to evaluate peri-implant tissues. It is not known if the ultrasound imaging settings might influence ultrasound’s ability to differentiate implant structures. The aim of this benchtop study was to evaluate the dependence of ultrasound on imaging angles and modes to measure implant geometry-related parameters.

**Methods:**

A clinical ultrasound scanner (ZS3, Mindray) with an intraoral probe (L30-8) offering combinations of harmonic and compound imaging modes was employed for imaging 16 abutments and 4 implants. The samples were mounted to a micro-positioning system in a water tank, which allowed a range of -30 to 30-degree imaging angles in 5-degree increment between the probe and samples. The abutment angle, implant thread pitch and depth were measured on ultrasound, compared to the reference readings. The errors were computed as a function of the image angles and modes. All samples were replicated 3 times for 3 image modes and 11 image angles, thus resulting in 2,340 images.

**Results:**

The mean errors of ultrasound to estimate 16 abutment angles, compared to the reference values, were between -1.8 to 2.7 degrees. The root mean squared error (RMSE) ranged from 1.5 to 4.6 degrees. Ultrasound significantly overestimated the thread pitch by 26.1 μm to 36.2 μm. The error in thread depth measurements were in a range of -50.5 μm to 39.6 μm, respectively. The RMSE of thread pitch and depth of the tested 4 implants was in a range of 34.7 to 56.9 μm and 51.0 to 101.8 μm, respectively. In most samples, these errors were independent of the image angle and modes.

**Conclusions:**

Within the limitations of this study, high-frequency ultrasound was feasible in imaging abutments and implant fixtures independent of scanning angle within ±30° of normal incidence and for compounding and non-compounding-based imaging modes.

## Introduction

Dental implants are the nearest equivalent replacement to missing teeth [1]. Despite their high survival rates, there is a pressing concern for losing peri-implant hard and soft tissues due to postoperative complications. The most common biological complications are mucositis and peri-implantitis. Mucositis is characterized by the appearance of inflammatory changes in the peri-implant mucosa that is clinically reversible after intervention [2]. Peri-implantitis is considered a bacterial pathogen-induced disease comparable to periodontitis, resulting in loss of the supporting bone and eventually the implant itself [3]. Swelling and erythema of peri-implant soft tissue, deeper probing depth, bleeding on probing, radiographic progressive bone loss, and implant mobility are all possible signs and symptoms [4]. Depending on the disease definition threshold, peri-implantitis is prevalent, affecting approximately 20 percent of implants [5]. Reported risk factors for peri-implantitis are patient-related factors, implant characteristics, implant location and clinician experience [6–8]. The current treatment strategies of these diseases include non-surgical and surgical therapy. Another disease category is soft and hard tissue deficiencies around implants that compromise function and esthetics [9]. Since there is currently no satisfactory treatment modality, prevention and early diagnosis of these diseases is critical [10].

The clinical standard for evaluating marginal bone loss is two dimensional (2D) intraoral radiographs. However, lack of cross-sectional information, image magnification/distortion and unavailability of facial/palatal/lingual bone levels are the major disadvantages [11]. Cone Beam Computed Tomography [CBCT] can provide 3D and cross-sectional information and is increasingly used to evaluate bone loss [12]. Repeated radiation exposure, higher cost, presence of artifacts around metallic implants and the inability to identify thin bone are its limiting factors [13, 14]. The status of peri-implant soft tissue is mainly evaluated by probing and the bleeding tendency. Although easy to perform, these procedures are not reliable and dissociated with the disease progression.

Ultrasonic imaging has been used extensively in quantitative medical diagnostics. In dentistry, it is primarily used as a research tool for evaluating tooth structure [15, 16], periodontal bony defects [17, 18], soft tissue lesions [19, 20], gingival thickness [21–23], and implant therapy [24]. It has also been reported that ultrasonic spatial measurements could potentially be a diagnostic test for diabetes mellitus [25]. A recent study revealed that ultrasound was accurate in determining alveolar bone level and its thickness [26], in addition to imaging oral anatomical structures [27, 28]. A recent systematic review revealed that using ultrasound imaging would be beneficial during planning, intraoperative and follow up phases of implant therapy [29]. A proof-of-principle preclinical study also suggested the feasibility of ultrasound in imaging peri-implant tissues [30]. With advantages of being non-ionizing and providing optimal soft-hard tissue interface contrast, it can be useful research as well as clinical imaging modality to examine and monitor peri-implant tissue health.

To evaluate peri-implant tissues, implant structures, including the implant fixture, abutment and prosthesis are important reference landmarks. Since ultrasonic imaging relies on sound reflection, visualization of these structures is influenced by the ultrasound frequency, which dictates image resolution, the scanning angle, and the scanning mode. High frequency ultrasound transducers can provide high image resolution and potentially improve interpretation of these implant structures. Implant structures act as specular reflectors and yield high amplitude, hyperechoic, signals. They commonly show reverberation artefacts and comet tails. Sound interfering with specular reflectors follows Snell’s Law and thus, depending on the geometric angle between the ultrasound beam and the reflecting surface, the returning sound may not be received by the probe. For large enough angles, the reflection off of the implant structure will become too weak and insufficient to form an image. Medically relevant specular reflectors such as vascular walls, soft tissue masses and others lead to the development of the advanced imaging technique compounding. Here, images are created from a range of internal angles with respect to the field of view. By superposition, i.e., compounding these images to one single image, the surface of specular reflectors can be visualized to a larger extent.

It is currently not known whether there is an optimal probe positioning angle and scanning mode in relation to the implant structures for ultrasound peri-implant tissue imaging. Therefore, the primary aim was to study the influence of scanning angles and modes on implant structure interpretation on ultrasound images.

## Materials and methods

### Experimental setup

**Fig 1:**
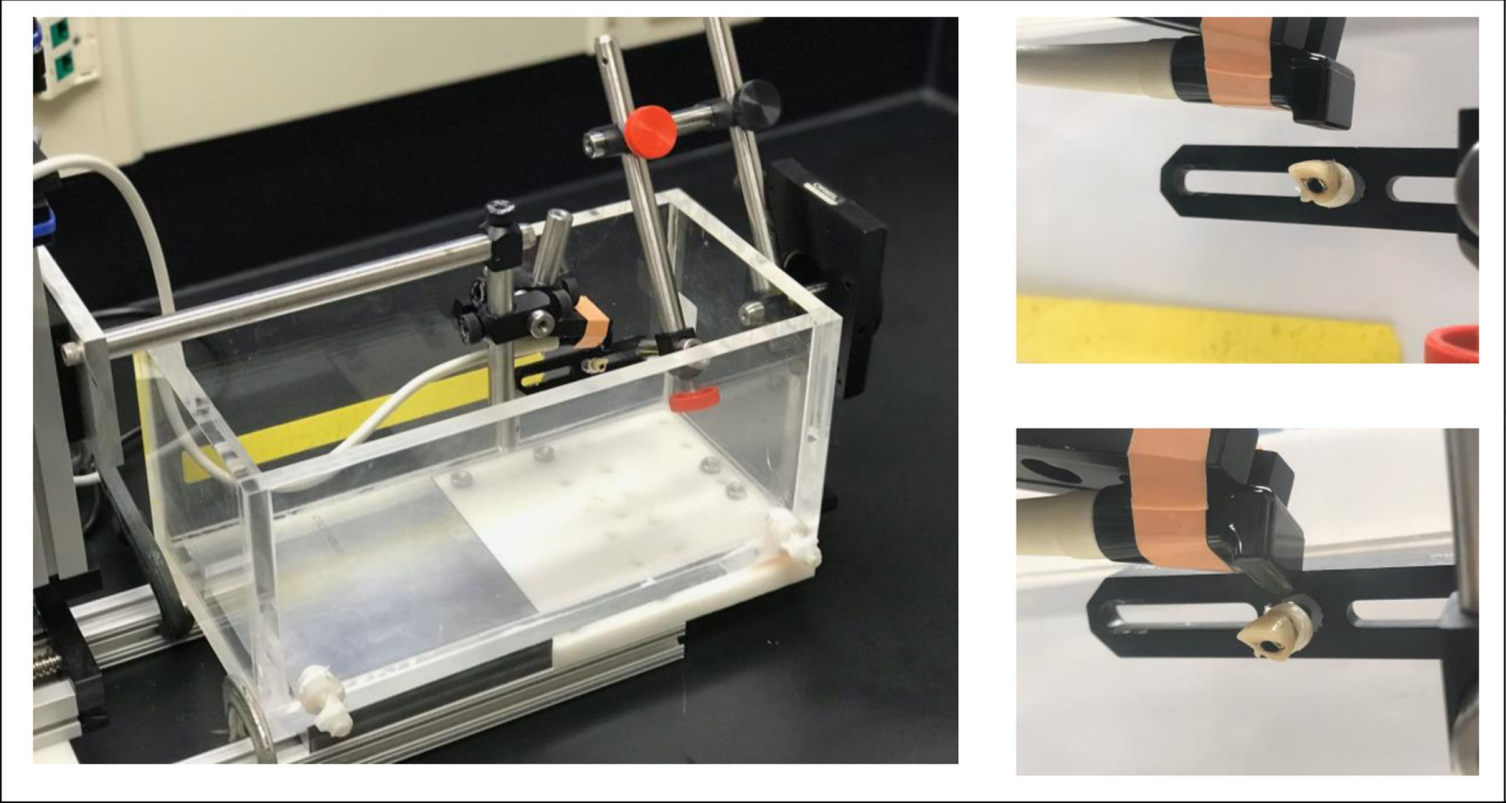
Experimental setup of this study. Left: (Empty) water tank to allow for acoustic coupling to the specimens, the rotational positioner, and the ultrasound probe. Right-top: Filled-water tank with imaging probe parallel (0°) with respect to the sample. In this image, a temporary crown is demonstrated. Right-bottom: The probe is at a large angle (+30°) with respect to the sample.

A series of 2 benchtop experiments (#1: implant abutments and #2: implant fixtures) were conducted to study the influence of scanning angles and ultrasonic imaging modes on characterizing implant abutment and fixture structures. Each of the implant abutments (N = 16) and fixtures (N = 4) were attached to a rotational micro positioner (RSA-1, Newport, Irvine, CA, USA) that rotated these specimens in relation to a fixed prototype linear array transducer (L30-8, previously labeled as L25-8, 24 MHz nominal imaging frequency, probe housing size of 30×18×12 mm, see Fig 1, left). The specimen was placed in a water tank to enable the scanning (Fig 1), and a commercial clinical ultrasound scanner (ZS3, Mindray of North America, Mountain View CA, USA) was used to record images.

**Fig 2:**
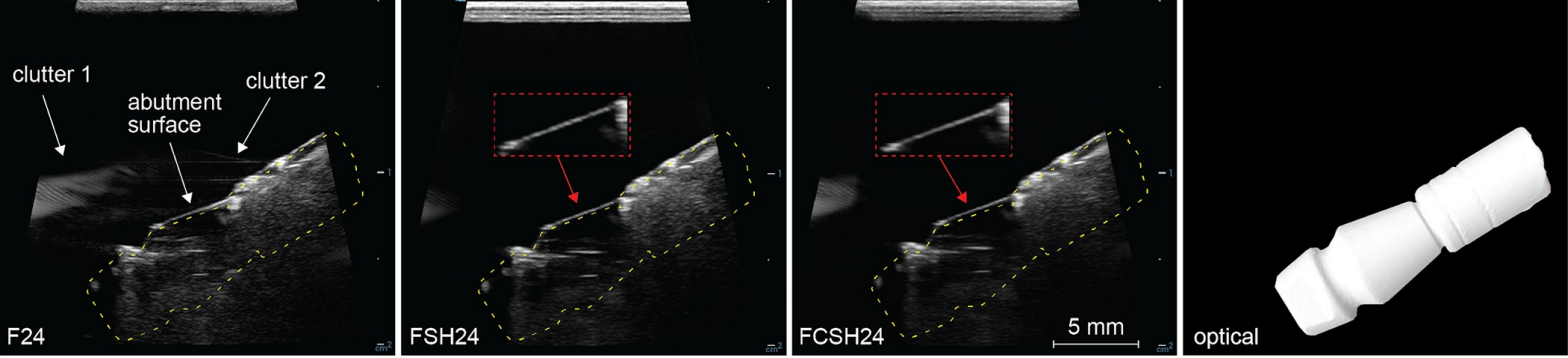
Ultrasonic images of an abutment to illustrate the difference between the investigated imaging modes, namely F24 (single frequency imaging), FSH24 (spatial harmonics), and FCSH24 (compound spatial harmonics). A yellow dashed outline of the abutment is shown within each ultrasound image. An optical scan of the same sample is shown in the right-side panel. Observable image differences include clutter reduction, which is mostly seen in F24 (clutter 1 and 2), much less in FSH24 and FCSH24. FCSH24 and FSH24 differ in the delineation of the abutment surface. This can be seen in the enlarged inset (red dashed frame), where FCSH24 shows a more continuous rendering of the abutment surface. These differences might influence imaging interpretation.

**Fig 3:**
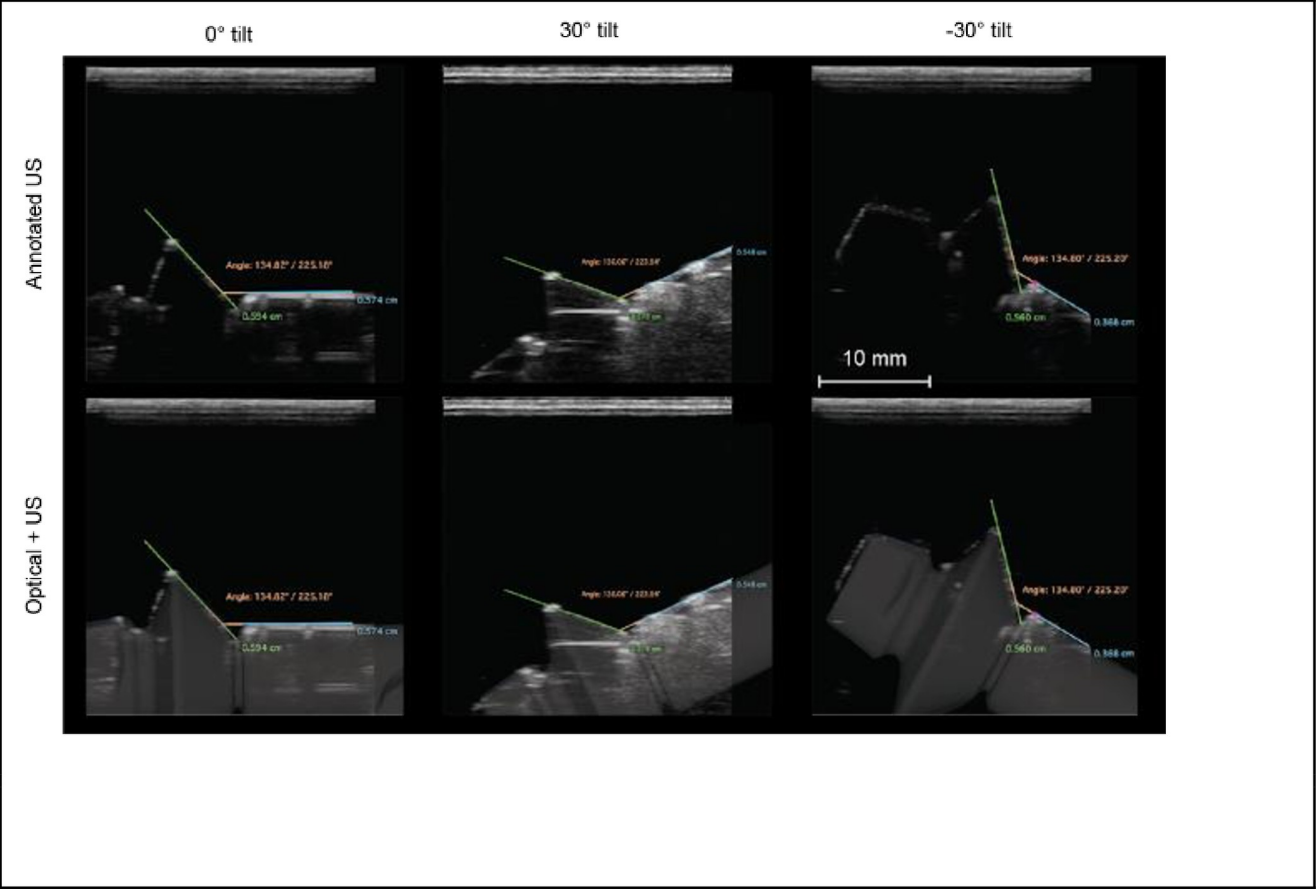
Top row: Measurement of the abutment angle on abutment 9–3. Columns left, middle and right, are labeled with the degree angle between the abutment and the ultrasound transducer, i.e., 0°, +30°, and -30° with respective abutment-holder angle measurements of 134.8°, 136.1°, and 134.8°. Bottom row: Overlay images for the respective optical (reference) and ultrasonic scans. (Scale bar for all images).

Spatial details of the abutments and fixtures were measured by ultrasound using 3 scanning modes, i.e., F24, FSH24 and FCSH24. Here FCSH24 stands for compound spatial harmonics imaging at 24 MHz with one fundamental frequency and two harmonic frequencies, FSH24 for spatial harmonics imaging at 24 MHz with three harmonic frequencies, and F24 for one single frequency imaging at 24 MHz (Fig 2). Maximum depth of scanning was set to 1.5 cm and the samples were placed in the center of the field of view. In addition, a gain of 60 [dB] was maintained. The in-plane angle of the probe was calibrated to the long-axis of the implant as such at 0° the ultrasound wave is propagated in perpendicular to the abutment holder or the implant fixture (see Fig 1 (right) and Fig 3). Once calibrated, i.e., zeroed with respect to angle, the sample was rotated in-plane between -30° and +30°, with steps of 5°, resulting in 13 images. A similar study was conducted previously using a mechanically swept single element transducer at 24 MHz [31]. At each angle 3 images were acquired for each of the 3 imaging modes used. The order of data acquisition was to acquire 3 imaging modes for a given sample and given tilt angle, repeat for 13 tilt angles, then switch to the next sample and repeat. After all samples were imaged, each sample was placed in the tank again to obtain a total of 3 independent assessments. This yields 13×3×3, i.e.117 images per sample and a total of 117×(16+4), i.e., 2,340 images for scanning of 16 abutments and 4 fixtures.

Spatial measurements were evaluated as the function of the specimens, scanning angles, and scanning modes. All the specimens were displayed on the ultrasound images as bright white reflections, i.e., hyperechoic echoes often with veils behind the reflection because of the internal acoustic reverberation. The images were then saved in both DICOM and JPEG formats for further interpretations.

### Samples and measurements

#### Experiment 1: Scanning of prefabricated implant abutments

Sixteen abutments (Neobiotech, South Korea) were used, consisting of sizes 3.5 mm diameter—3 mm height to 9 mm diameter—4 mm height, to provide a variety of abutment emergence angles. The primary measurement was the angle between the abutment surface and the attached abutment holder.

*Ultrasonic assessment*. Ultrasound images in DICOM format were analyzed by one calibrated examiner (NG) using open-source software with built-in calipers (Horos, The Horos Project & OsiriX Team, version 3.3.6). Intra-examiner calibration was performed by measuring all parameters on 10 randomly selected implants and abutments repeatedly, at least one day apart to achieve an intra-examiner agreement of at least 0.8. Fig 3 shows 3 example images of abutment 9–3. In the left side panel, the abutment-holder was positioned such that the holder formed an angle of 0° with respect to the transducer aperture (i.e., the horizontal). As an additional example, the middle panel shows a setup with an angle of +30°. In this case the abutment-holder structure tilted upward from left-to right, which we arbitrarily defined as a positive angle. The right-side panel shows a setup with an angle of -30°. The abutment angle was determined by drawing two horizontal lines along the planes of the hyperechoic reflections of the abutment and holder surfaces. The angle formed by the virtual intersection was assessed.

*Optical assessment (Reference)*. The samples were scanned using an intraoral scanner (TRIOS 3, 3Shape A/S, Copenhagen, Denmark) to generate standard tessellation language (STL) files. STL files were opened with the built-in software, where cross-sectional images at the mid aspect of the implant-abutment-crown structure were obtained. These cross-sectional images were calibrated by means of another software (ImageJ public domain, NIH, imagej.nih.gov) by a calibrated examiner (RS), using the known implant diameter as measurement reference. S1 Fig shows the obtained 3D depictions of all 16 samples in the order of left to right (sample 1, 2, 3, and 4), then top to bottom (samples 5 to 8, etc.). Results of the optical measurements are shown in columns 4 to 6 of S1 Table. The mean and coefficient of variation in percent are shown in columns 7 and 8. The maximum coefficient of variation is 3.6%, the mean 0.97%. Note that S1 Table lists the complement angles for abutments 3.5–3, 3.5–5, and 4–3.

#### Experiment 2: Scanning of implant fixtures

Four implants (Neobiotech, Korea) consisting of sizes 3.5 mm (diameter) -13 mm (length), 4.0–13, 4.5–13 and 5–13 were scanned by the same intraoral scanner and ultrasound devices. S2 Table provides results of 5–6 measurement points of thread depth per implant (column 4 to column 8), as well as mean and coefficient of variance. Thread pitches were available from the manufacturer’s manual as 0.8 mm for all four implants.

**Fig 4:**
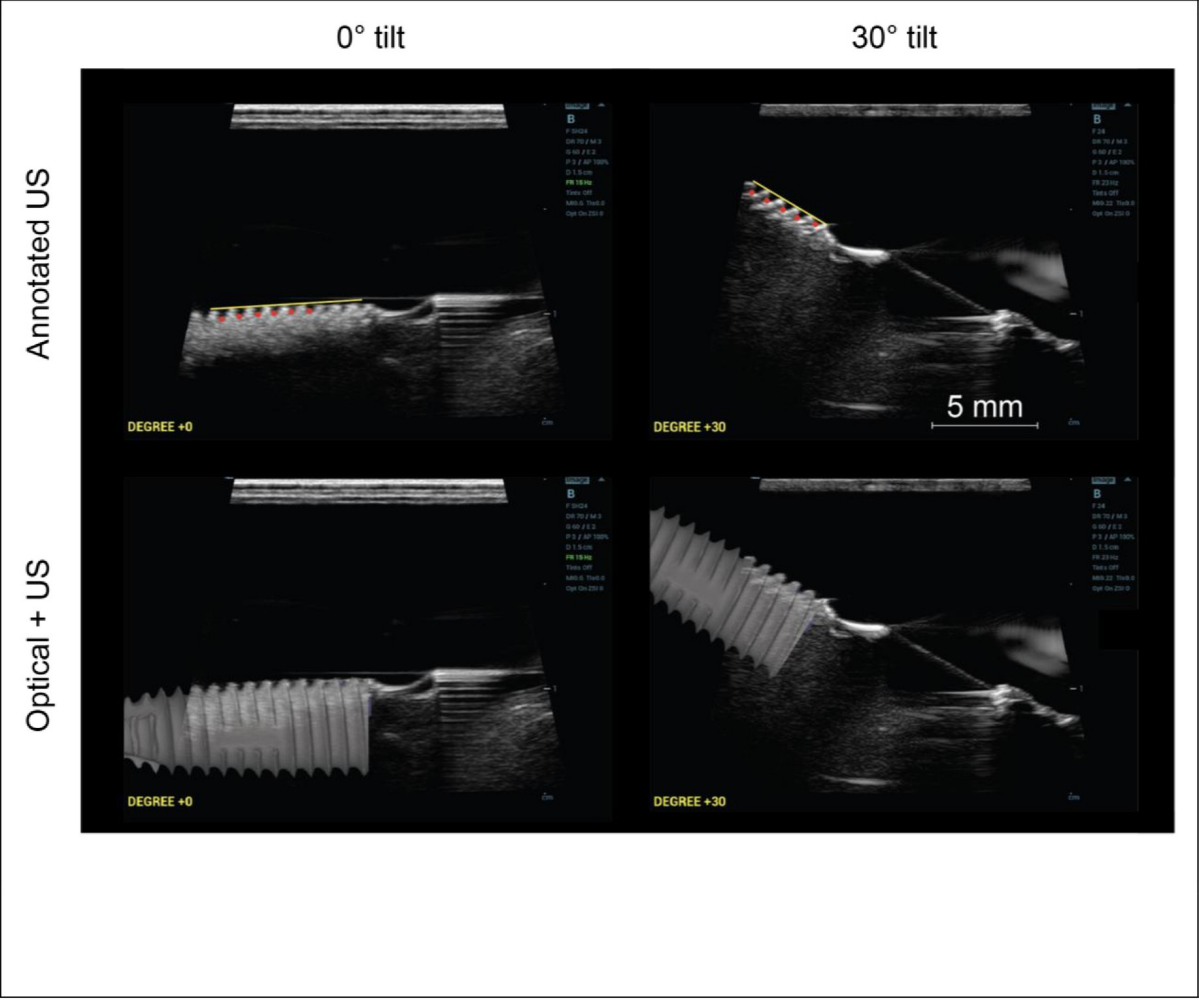
Implant thread pitch and thread depth measurement dependence on the underlying image angle, i.e., angle between the implant and ultrasound array, e.g., DEGREE +0 or +30 in the shown images. Top: A line connecting the peaks of at least 5 consecutive threads is drawn (yellow line). Associated valleys are identified and shown as red dots. The thread depth was measured from the yellow line to each individual red dot and averaged. The thread pitch was measured as the distance between the adjacent red dots that were projected to the yellow line and averaged. Bottom row: Overlaid semi-transparent optical scan of the imaged implant, i.e., a 4.5–13 as shown in S2 Fig. The scale bar is for all images.

*Ultrasonic assessment*. Fig 4 shows two example images of implant 4.5–13 for two different image angles, i.e., 0° and +30°. In the left side panel, the implant structure was positioned such that it formed an angle of 0° with respect to the transducer aperture, i.e., the horizontal. Whereas the right-side panel shows the implant structure at an image angle of +30°. Threads are visible in a continuous periodic manner, as peaks and valleys. Thread pitch and thread depth were derived by placing a straight reference line (the yellow line in Fig 4) connecting the thread peaks. Five perpendiculars were dropped at five consecutive valley locations (identified and marked with red dots) along the reference line. The incremental distance between these positions gives the thread pitch and the distance between the straight line and the valley points give the thread depth. The average pitch and depth were acquired for each image. Measurements were performed for all image angles from -30° to +30° in steps of 5° as well as the 3 imaging modes.

### Statistical analysis

**Fig 5:**
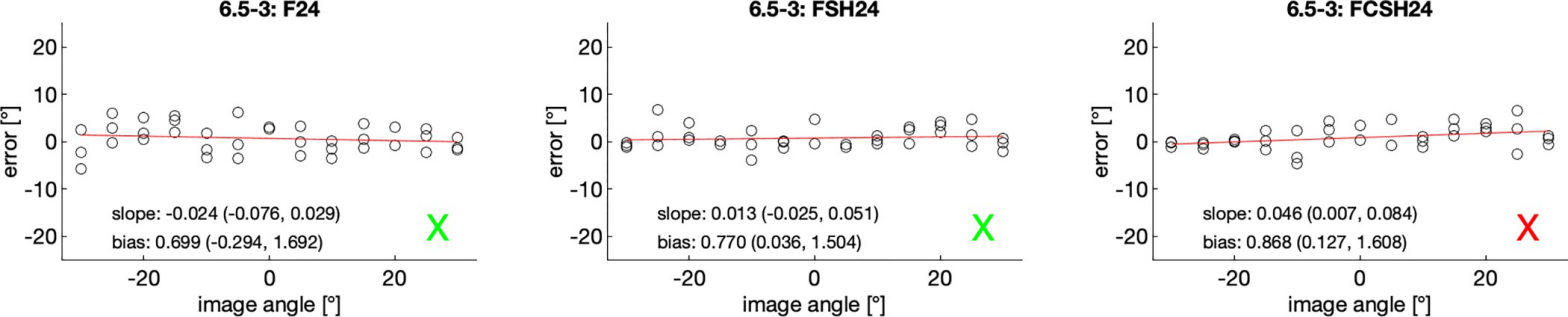
Error in measuring abutment angle using ultrasonic imaging as a function of image angle. In this particular example, abutment type 6.5–3 was measured using 3 imaging modes, i.e., F24, FSH24, and FCSH24, depicted above from left to right. For each imaging mode the image angle was arranged from -30° to +30° and three estimates of the abutment angle were taken. The resulting error was then fit using a 1^st^ order polynomial. Slope and bias with 95% confidence interval are provided for each imaging mode. A red ‘X’ indicates a slope being significantly different from zero; and ‘X’ is vice versa.

In experiment 1, the degree-error (the difference between the ultrasound measurement and the reference measurement) was plotted as a function of the scanning angle and fitted to a linear regression (y = a×x+b), thus resulting in a slope (a) and bias (b) with 95% confidence interval. If the 95% confidence interval of the slope contained zero, the degree-error was determined as independent of the scanning angle. The bias of ultrasound measurements for each of the 3 scanning modes was also calculated. An example is given in Fig 5, showing the degree-error for assessing abutment sample 6.5–3, which was computed as measured angle (ultrasonically) minus the true angle (optical assessment, see S1 Table). Three imaging modes, namely F24, FSH24, and FCSH24, were used and results for each are shown in the left, middle and right panels, respectively. In cases when the slope was not statistically different from 0, a green ‘X’ was placed to the right of the slope/bias information. If, however, the slope was determined as a function of the scan angle, then a red ‘X’ was placed.

The root mean squared error (RMSE) was calculated for each of the 16 abutments and each of the 3 scanning modes. In experiment 2, the implant thread pitch and depth error were again plotted as a function of the scanning angle and fitted to a linear regression in the same manner as experiment 1. The bias and slope, as a result of different scanning angles and scanning modes, were also calculated.

## Results

### Experiment 1

**Fig 6:**
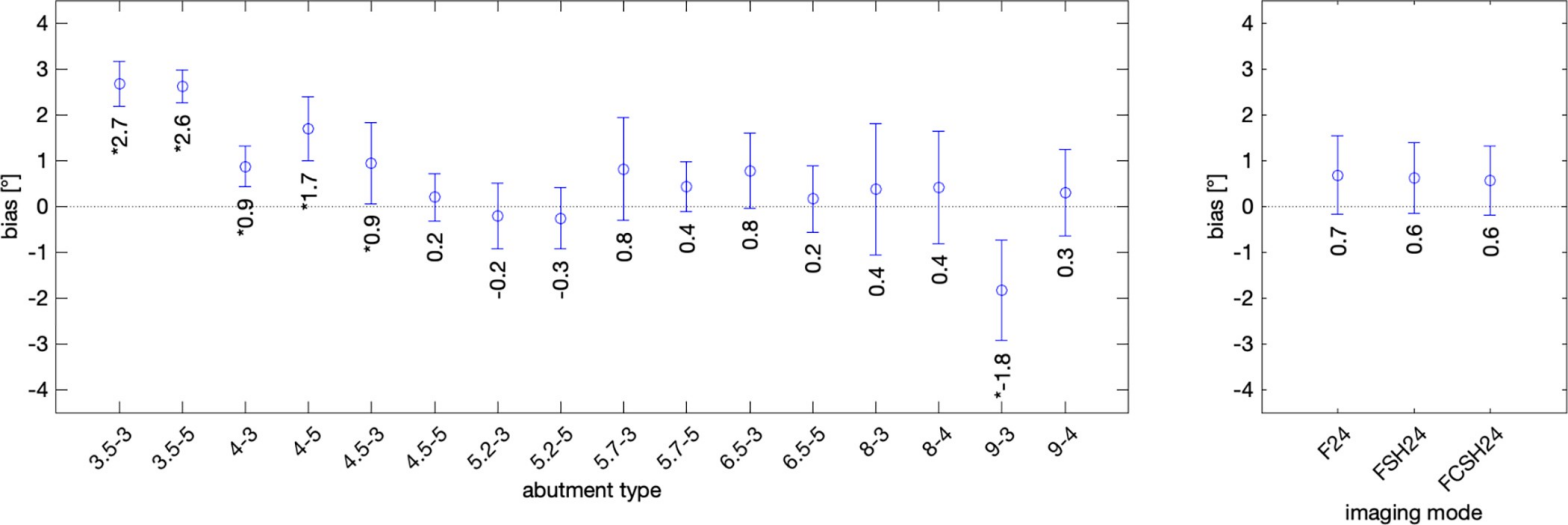
Bias in measuring abutment angles, for all 16 abutments. Left graph: The mean bias in degrees and confidence interval error bars across three imaging modes (F24, FSH24, FCSH24). Most of the biases, even if statistically significant, were small. Right graph: Mean bias in degrees and confidence interval error bars across 16 abutment types (3.5–3 to 9–4) for each imaging mode (F24, FSH24, and FCSH24).

An overview of ultrasound angle measurement errors of all 16 abutments is given in S3 Fig. Fig 6 summarizes the mean bias for each sample (left panel). Confidence interval (95%) error bars indicate the level of repeatability. Data in the left panel was averaged across all three imaging modes. A few samples show small and clinically negligible bias yet with statistical significance. Whereas the right panel also shows mean bias, but averaged across all 16 samples, instead here the dependence of the estimate with respect to the imaging mode was evaluated. Among the three imaging modes, there is no appreciable difference.

**Fig 7:**
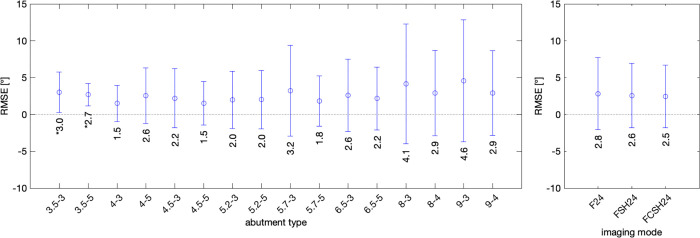
Left graph: Root mean squared error (RMSE) in measuring abutment angles of the 16 specimens. The RMSE is the range of 1.5 to 4.6 degrees. Right graph: Mean RMSE in degrees and confidence interval error bars across 16 abutment types for each imaging mode (F24, FSH24, and FCSH24). The RMSE is in the range of 2.5 to 2.8 degrees for all 3 modes without statistical significance.

Fig 7 shows RMSE of the tested samples. The remainder of the Figure is analogous to Fig 6. The RMSE is in a range of 1.5 to 4.6 degrees, without statistical difference from 0, except for sample 3.5–3. Assessing the performance of imaging mode shows an average error or less than 5°. Most of the estimates (left and right graph) show a confidence interval of approximately ±2.5°.

**Fig 8:**
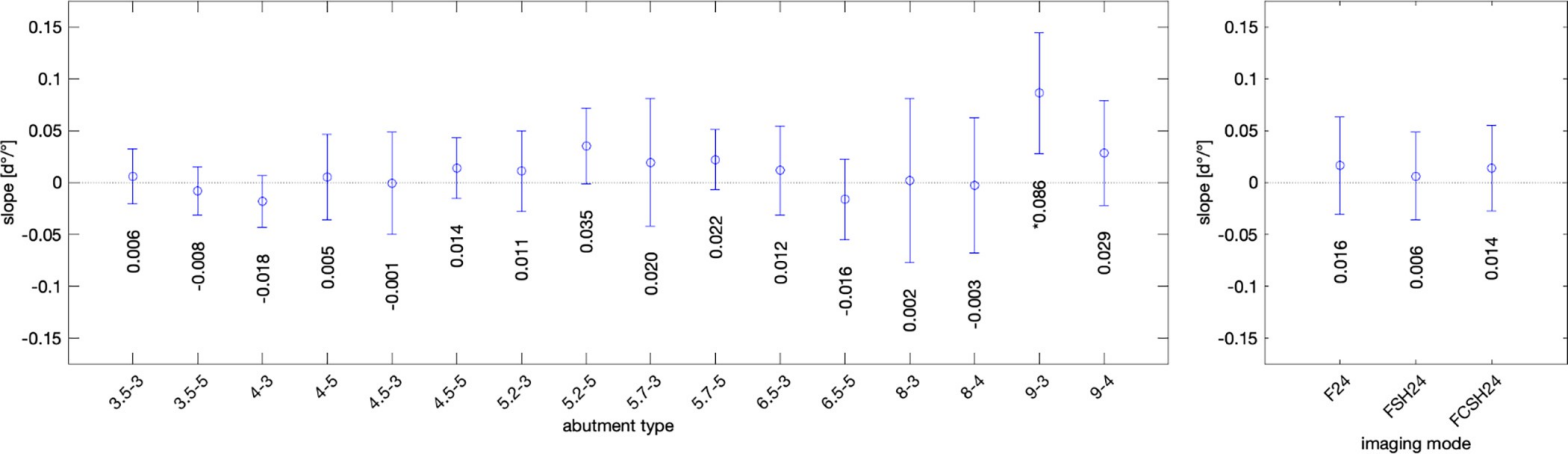
Abutment angle measurement dependence on the scanning angle. Left graph: Summary statistics for data in S3 Fig. Mean slope across 3 imaging modes (F24, FSH24, FCSH24) is depicted in units of error in abutment angle measurement (d°) divided by image angle in units of degree (°), i.e., d°/°. Error bars present the confidence interval across F24, FSH24, FCSH24. Data is shown for all 16 abutment types listed in S1 Table. Right graph: Mean slope and standard deviation across 16 abutment types for each imaging mode (F24, FSH24, and FCSH24). The slope is negligible, suggesting the error is independent of the tested scanning angle.

Fig 8 evaluates the functional dependence of error due to the scanning angle. Mean slope across three imaging modes and three repeat-measurements is in units of error in abutment angle measurement (degree) divided by the image angle (in degree). Error bars are based on one standard deviation. The largest slope is shown for sample 9–3 with an average of almost 0.1°/°. Practically this means that over the interval of ±30° tilt angle, a ±3° variation of the abutment angle was seen. Most samples indicate a positive slope, i.e., there was a preferred direction, however the magnitude is small. The right panel shows the dependence of slope with respect to the imaging mode and no appreciable dependence is seen.

### Experiment 2

**Fig 9:**
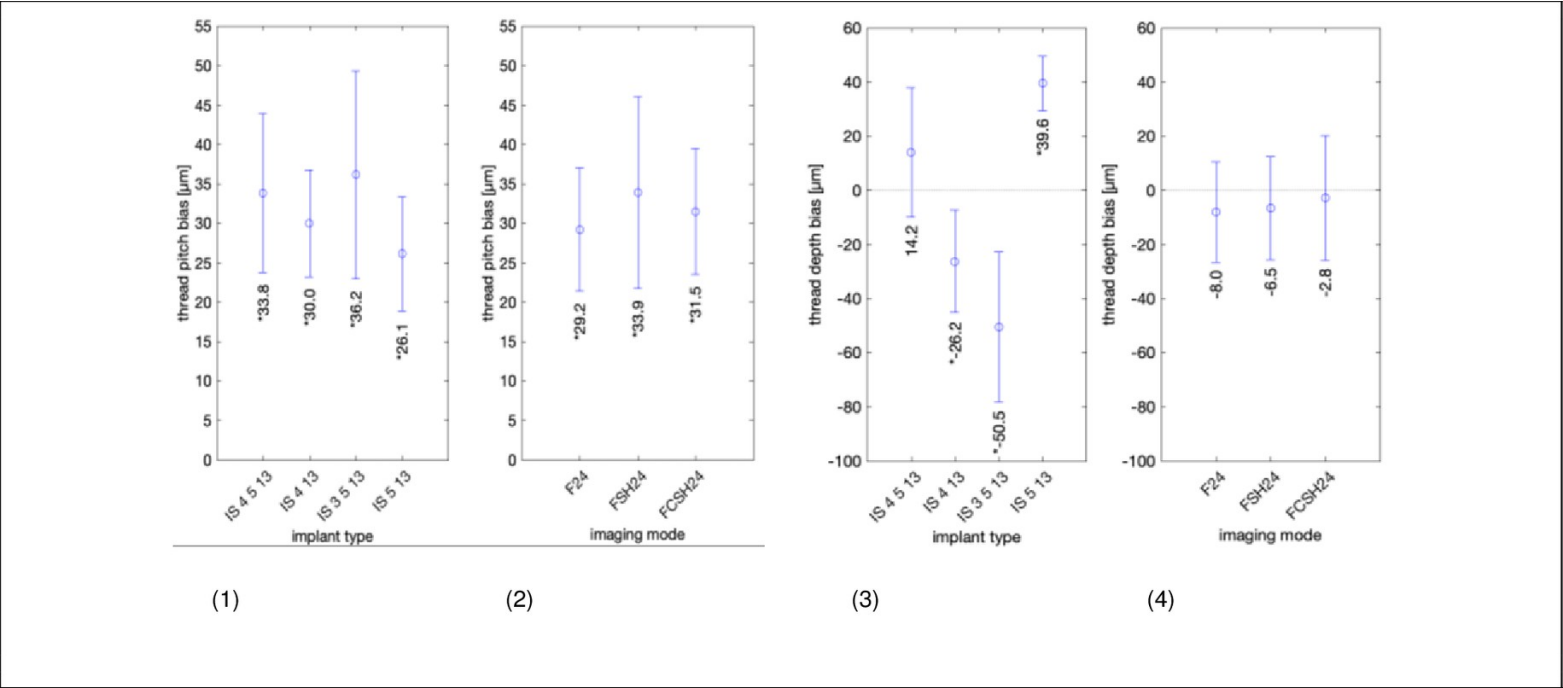
Bias and dependence of measuring implant thread pitch (left) and thread depth (right) with ultrasound. Left to right: (1) Bias in micrometer with 95% CI for each of the 4 implant samples. (2) Bias in micrometer 95% CI for the 3 imaging modes (F24, FSH24, and FCSH24). (3 and 4) Same data as (1 and 2) but showing thread depth bias in micrometers. The complete data are shown in S4 and S5 Figs, for implant thread pitch and thread depth, respectively.

The error in measuring implant thread pitch and depth using ultrasound is provided in Fig 9, S4 Fig and S5 Fig, respectively. Statistically significant mean overestimation of thread pitch of 26.1 to 36.2 μm was found (Fig 9-1). The mean bias in estimating thread depth ranges from -50.5 μm to 39.6 μm (Fig 9-3).

**Fig 10:**
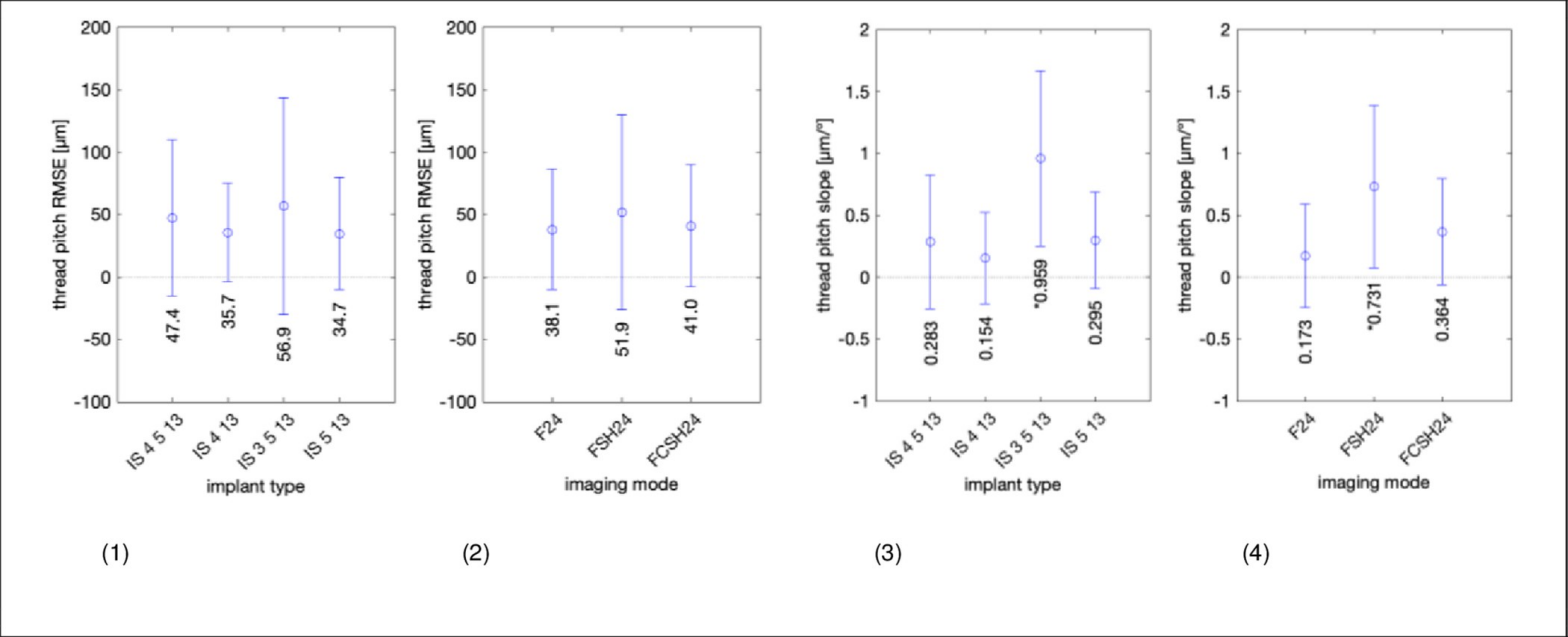
Root mean squared error (RMSE) and image angle dependence of measuring implant thread pitch with ultrasound. Left to right: (1) RMSE in micrometer with 95% CI error bars for each of the 4 implant samples. (2) Mean error in micrometer and 95% CI error bars for the 3 imaging modes (F24, FSH24, and FCSH24). (3 and 4) Same data as (1 and 2) but as dependence of image angle, i.e., the slope in each subplot combination of implant and imaging mode. The complete data are shown in S4 Fig.

Fig 10 (left panel) shows RMSE for ultrasound thread pitch measurements with 95% CI. All pitch RMSE errors are less than 20% of the 800 μm true pitch, i.e., less than 160 μm, with standard deviations of up to 10%. The second panel shows RMSE for the three imaging modes. The RMSE is approximately 5% for the 3 modes. The next two panels use the same scheme to display the slope of the thread pitch error (in % per degree). All slopes are less than 0.1%/°, except for sample IS 3.5–13, which was also significantly dependent on the image angle. The imaging mode FSH24 also shows the largest dependence of 0.09%/°.

**Fig 11:**
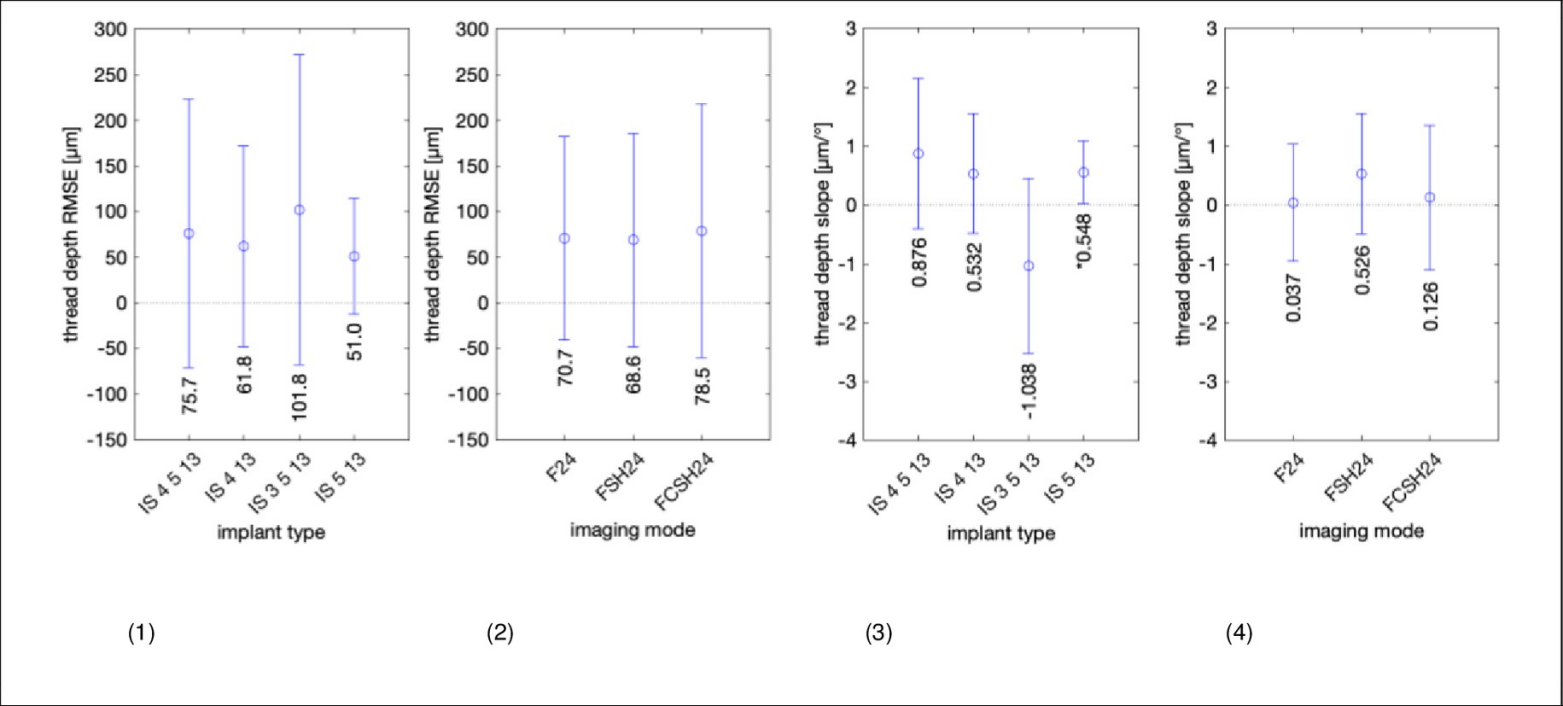
RMSE in measuring implant thread depth with ultrasound. Left to right: (1) RMSE in micrometers and standard deviation error bars across 3 imaging modes (F24, FSH24, FCSH24) for each of the 4 implant types. (2) RMSE in micrometers and standard deviation error bars across 4 implant types for each of the 3 imaging modes (F24, FSH24, and FCSH24). (3 and 4) Same data as (1 and 2) but as dependence of image angle, i.e., the slope in each subplot combination of implant and imaging mode of S5 Fig.

Fig 11 shows RMSE of thread depth measurements with 95% CI. The largest RMSE was found for implant 3.5–13 (101.8 μm) with a confidence interval of ±170 μm. All three imaging modes show approximately the same error of 70 to 80 μm with a standard deviation of ~±130 μm, as can be seen in panel 2. The next two panels display the slope of the thread depth error (in μm per degree). All errors are between ±1.0 μm/°. Implant 3.5–13 shows the largest dependence of -1.0 μm/° and imaging mode FSH24 also shows the largest dependence of 0.526 μm/°.

## Discussion

### General findings and clinical implications

Peri-implant diseases, especially peri-implantitis, are becoming the main threat to the long-term function and esthetics of dental implants. Clinical strategies to halt disease progression and implant loss largely remain ineffective [32]. Therefore, prevention and early diagnosis/intervention become critical for the longevity of dental implants. Ultrasound as a promising imaging modality for peri-implant tissue evaluation has recently been documented in the literature [30, 33–35]. This study further investigates the potential influence of scanning angles and modes on interpretation of implant structures. This study indicates that within ±30-degree scanning angle the ability to identify abutments and implant fixtures on ultrasound images is independent of the scanning angle. This gives ultrasound operators some freedom in capturing implant structures, e.g., abutment surface and implant platform that are primarily used to quantify peri-implant hard and soft-tissue structures. In the same token, the 3 tested imaging modes did not have a noticeable influence on depicting abutments and implants. Whether these findings may also apply with presence of a soft tissue layer in the clinical scenario deserves further investigation.

This study shows clinical accuracy of high-frequency ultrasound to measure the abutment angle, with the absolute error ranging between 2 to 5 degrees across an array of the tested abutments with various emergence angles (Fig 7). Clinically, the emergence angle of dental implants varies significantly, depending on the implant position and crown size, etc. [36] Emergence angle may be associated with peri-implant tissue dimension and mucosal margin location [34, 37]. Therefore, ultrasound can be useful to evaluate the emergence angle of abutments non-invasively before making clinical decisions. This study also shows clinical accuracy of high-frequency ultrasound to imaging implant thread pitch and depth, with absolute errors of approximately 60 μm (7.1% of 800 μm) and 50 to 100 μm, respectively (Figs 10 and 11, respectively). Thread pitch and thread depth differ acoustically. Thread pitches are freely accessible by the ultrasound beam, whereas the thread depth is acoustically hidden by the neighboring thread walls (spaced by 800 μm). Thread depth determination will suffer from acoustic clutter filling the trough, thus producing a larger variation with an increased RMSE. The spatial resolution of the ultrasound system is dictated by the employed transmit (TX) and receive (RX) frequencies. For TX/RX equal 12/24, i.e., imaging modes FSH24 and FCSH24, the inherent spatial resolution is 128/64, i.e., 96 μm. For TX/RX equal 24/24, i.e., imaging modes F24, the inherent spatial resolution is 64/64, i.e., 64 μm. These are approximate numbers. Note that the former two modes, FSH24 and FCSH24, use additional image formation and processing strategies to improve resolution and contrast, which are beyond the scope of this investigation.

Clinically, peri-implant bone remodels during initial healing, depending on the implant designs and the depth of implant placement in relation to the crestal bone level. The amount of this physiologic remodeling may be up to 2 mm approximately. The higher limit of bone remodeling may be found when implants with wider smooth-collar are used and placed equal-crestally or sub-crestally. Pathologic bone remodeling occurs with exuberant inflammation induced primarily by pathogenic bacteria and possible foreign body reaction, resulting in pronounced bone loss and exposure of implant threads beyond physiologic remodeling. Therefore, given the current finding that ultrasound could differentiate implant threads, it could inform the amount of peri-implant bone loss, thus potentially a real-time, and non-radiation imaging modality to evaluate peri-implant health in the clinic.

### Clinical scanning recommendations

Ideally, the sound propagation direction should be in perpendicular to the implant structures to achieve the largest sound reflection and to generate the veil of the implant structure on ultrasound images [38]. The veil is a good indicator of highly (acoustic) reverberating structures, e.g., implant surface, abutment, and crowns. However, implant and abutment axes are in most instances different in the cross-sectional view. Therefore, to see both structures in a single B-mode image, the operator shall identify one structure, e.g., the abutment surface first, followed by rotating the probe axially to find the implant fixture, or vice versa. In cases of healthy implants with minimal bone remodeling, it is easier to image the abutment surface, which has a larger dimension. In cases with pronounced bone loss, imaging the implant threads may become easier and should be pursued first. Additionally, in the oral cavity it is possible that the local geometry, e.g., vestibular depth and tongue, constrains the way the ultrasound probe can be positioned and therefore it is not always possible for the operator to place the ultrasound probe in the optimal direction. Based on the results of this current investigation, an ultrasound operator may have a freedom of at least ±30 degrees of the scanning angle to locate the abutment and implant fixture without compromising image quality.

### Study limitations

Major limitations of this study include (1) exclusive use of a single implant system, (2) only a single examiner to perform measurements, and (3) absence of an overlying soft tissue layer. Nevertheless, given the similarities in implant designs among major manufacturers and a wide geometry of abutments used in this study, the results should be generalizable. The examiner was self-calibrated and calibrated with the gold standard. However, multiple readers can give additional information, e.g., intra-class correlation coefficient and reader variations. Finally, subsequent studies with inclusion of soft tissue on top of implant structures are needed to mimic clinical scenarios. This parametric study serves to provide first quantitative evidence that ultrasound may image implant structures independent of the selected scanning angles and imaging modes.

## Conclusions

Within the limitations of this study, high-frequency dental ultrasound is feasible in imaging abutments and implant fixtures independent of scanning angle within ±30° of normal incidence and for compounding and non-compounding-based imaging modes.

## Supporting information

S1 Fig Reference standard estimation of the abutment angle for the 16 abutment types. Optical images of the 16 tested abutments attached to the holder, shown in S1 Table.
(DOCX)

S2 Fig Optical images of the 4 implant samples with the diameters (the 1^st^ number is the implant diameter, and the 2^nd^ number is the length in mm). (DOCX)

S3 Fig Overview of all abutment angle measurements for all 16 abutment types listed in S1 Table. Here, analogous to Fig 5, each plot figure also provided information on slope, bias, and image tilt angle dependence. Samples were grouped in sections of 3, corresponding to the 3 imaging modes. There were 8 rows and 2 columns of graphs. Abutment samples 1 to 8 from S1 Table were in the first column and the remaining samples in column two. To obtain a general understanding of the data shown in S3 Fig, statistical summary graphs were generated, shown here in Figs 6–8. See Fig 5 for a detailed description of a single case. See Figs 6–8 for statistical analysis of these measurements. **Note:** F24, -15° is an example for an erroneous reading due to limited field of view.
(DOCX)

S4 Fig Error in measuring implant thread pitch using high frequency ultrasonic imaging. Absolute error (μm) is given as a function of image angle between the implant and the ultrasonic imaging array. Optical measurements (see S2 Fig) serve as a reference standard. Four implant types, i.e., 4.5–13, 4–13, 3.5–13 and 5–13 (left to right) were assessed using 3 imaging modes, i.e., F24, FSH24, and FCSH24 (top to bottom). For each combination of implant and imaging mode the image angle was arranged from -30° to +30° and three estimates of the implant thread pitch were obtained. The difference between the ultrasonic and the optical measurement is plotted as the function of image angle. Finally, the resulting data is fitted using a 1^st^ order polynomial. Slope and bias are provided for each imaging mode. Slope describes dependence of the error with respect to the image angle. Bias describes any constant over- or underestimation of the measurement. For both, the 95% confidence interval is provided in parentheses. A green ‘X’ indicates a slope estimate for which the 95% confidence interval includes zero, i.e., the slope is not significantly different from zero.
(DOCX)

S5 Fig Error in measuring implant thread depth using high frequency ultrasonic imaging. Absolute error (μm) is given as a function of image angle between the implant and the ultrasonic imaging array. Optical measurements (see S2 Fig) serve as a reference standard. Four implant types, i.e., 4.5–13, 4–13, 3.5–13 and 5–13 (left to right) were assessed using 3 imaging modes, i.e., F24, FSH24, and FCSH24 (top to bottom). For each combination of implant and imaging mode the image angle was arranged from -30° to +30° and three estimates of the implant thread depth were obtained. The absolute difference between the ultrasonic and the optical measurement is plotted as the function of image angle. Finally, the resulting data is fitted using a 1^st^ order polynomial. Slope and bias are provided for each imaging mode. Slope describes dependence of the error with respect to the image angle. Bias describes any constant over- or underestimation of the measurement. For both, the 95% confidence interval is provided in parentheses. A green ‘X’ indicates a slope estimate for which the 95% confidence interval includes zero, i.e., the slope is not significantly different from zero.
(DOCX)

S1 Table Listing of optical abutment angle reference measurements (N = 3 per abutment). A total of 16 abutments were tested. Note: Abutments are labeled using the smaller angle, i.e., either the opening angle or its complement. Here, for abutments 3.5–3, 3.5–5, and 4–3, the complement angles are listed.
(DOCX)

S2 Table Listing of optical implant reference thread depth measurements (5 estimates per implant, with 3 repetitions each). True thread pitch (last column) was obtained from the manufacturer specifications.
(DOCX)
