# Supplementary material for: Ultrasound insonation angle and scanning imaging modes for imaging dental implant structures: A benchtop study
Source: PLoS One. 2022 Nov 29;17(11):e0270392. doi: 10.1371/journal.pone.0270392 (PMC9707752; doi:10.1371/journal.pone.0270392)
Supplement: S1 Fig — Optical images of the 16 tested abutments attached to the holder, shown in S1 Table. (DOCX) [file pone.0270392.s001.docx]

| 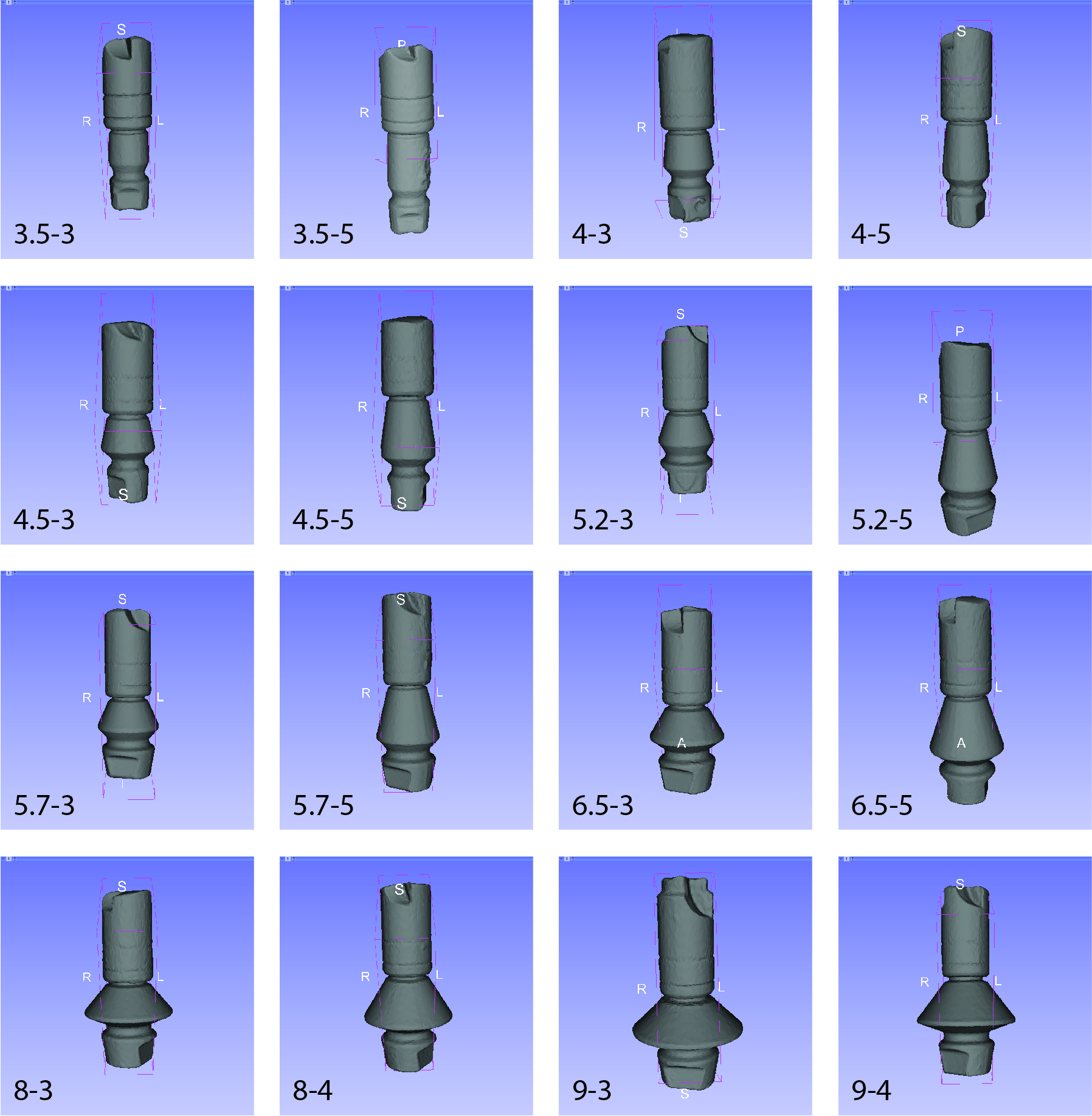 |
| --- |
|  |
| **Supplemental Figure S1.** Reference standard estimation of the abutment angle for the 16 abutment types. Optical images of the 16 tested abutments attached to the holder, shown in Supplement Table S1. |
