# Supplementary material for: Ultrasound insonation angle and scanning imaging modes for imaging dental implant structures: A benchtop study
Source: PLoS One. 2022 Nov 29;17(11):e0270392. doi: 10.1371/journal.pone.0270392 (PMC9707752; doi:10.1371/journal.pone.0270392)
Supplement: S2 Fig — (DOCX) [file pone.0270392.s002.docx]

| 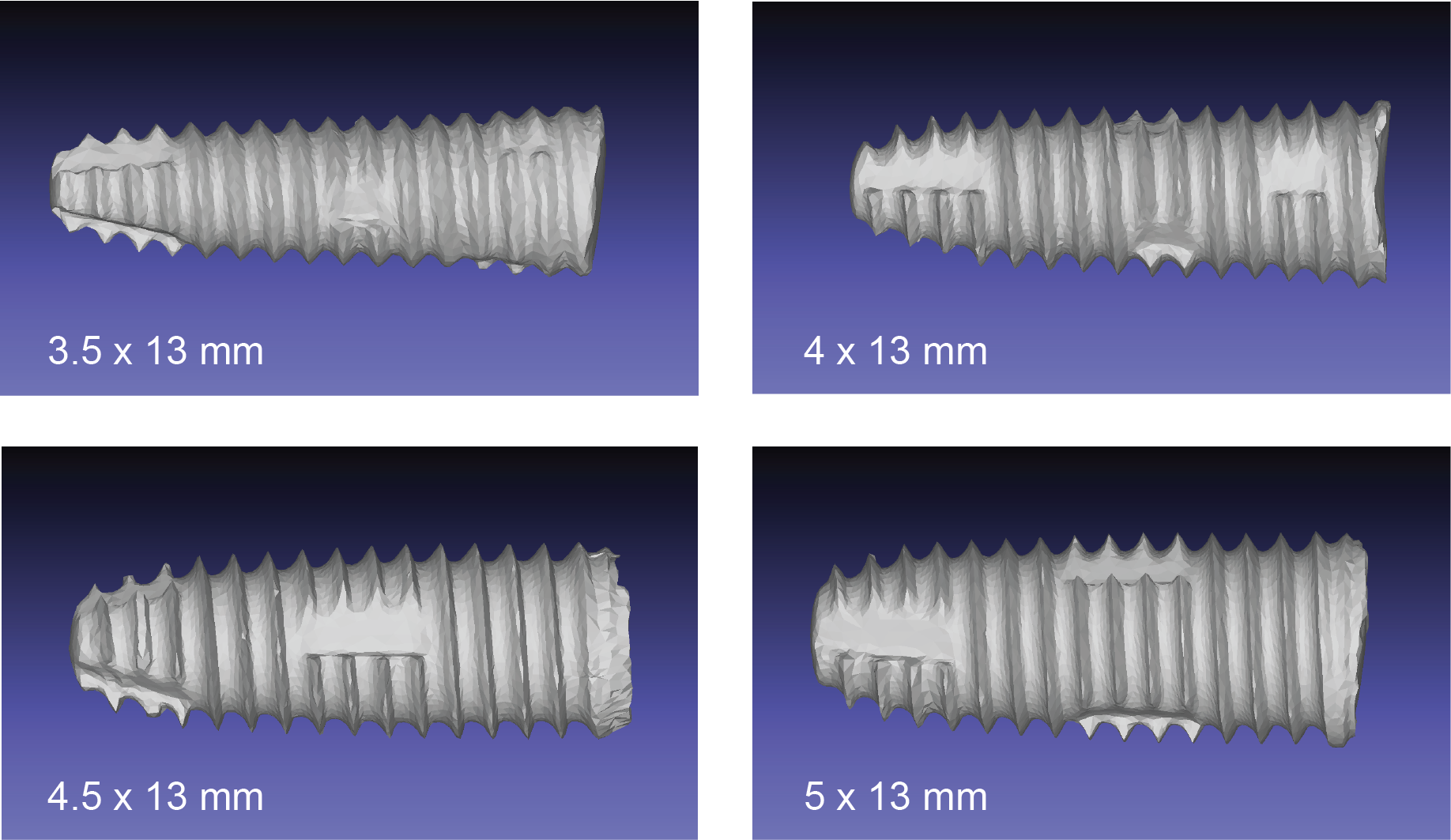 |
| --- |
|  |
| **Supplemental Figure S2.** Optical images of the 4 implant samples with the diameters (the 1^st^ number is the implant diameter, and the 2^nd^ number is the length in mm) |
