# Supplementary material for: Ultrasound insonation angle and scanning imaging modes for imaging dental implant structures: A benchtop study
Source: PLoS One. 2022 Nov 29;17(11):e0270392. doi: 10.1371/journal.pone.0270392 (PMC9707752; doi:10.1371/journal.pone.0270392)
Supplement: S3 Fig — Here, analogous to Fig 5, each plot figure also provided information on slope, bias, and image tilt angle dependence. Samples were grouped in sections of 3, corresponding to the 3 imaging modes. There were 8 rows and 2 columns of graphs. Abutment samples 1 to 8 from S1 Table were in the first column and the remaining samples in column two. To obtain a general understanding of the data shown in S3 Fig, statistical summary graphs were generated, shown here in Figs 6–8. See Fig 5 for a detailed description of a single case. See Figs 6–8 for statistical analysis of these measurements. Note: F24, -15° is an example for an erroneous reading due to limited field of view. (DOCX) [file pone.0270392.s003.docx]

| 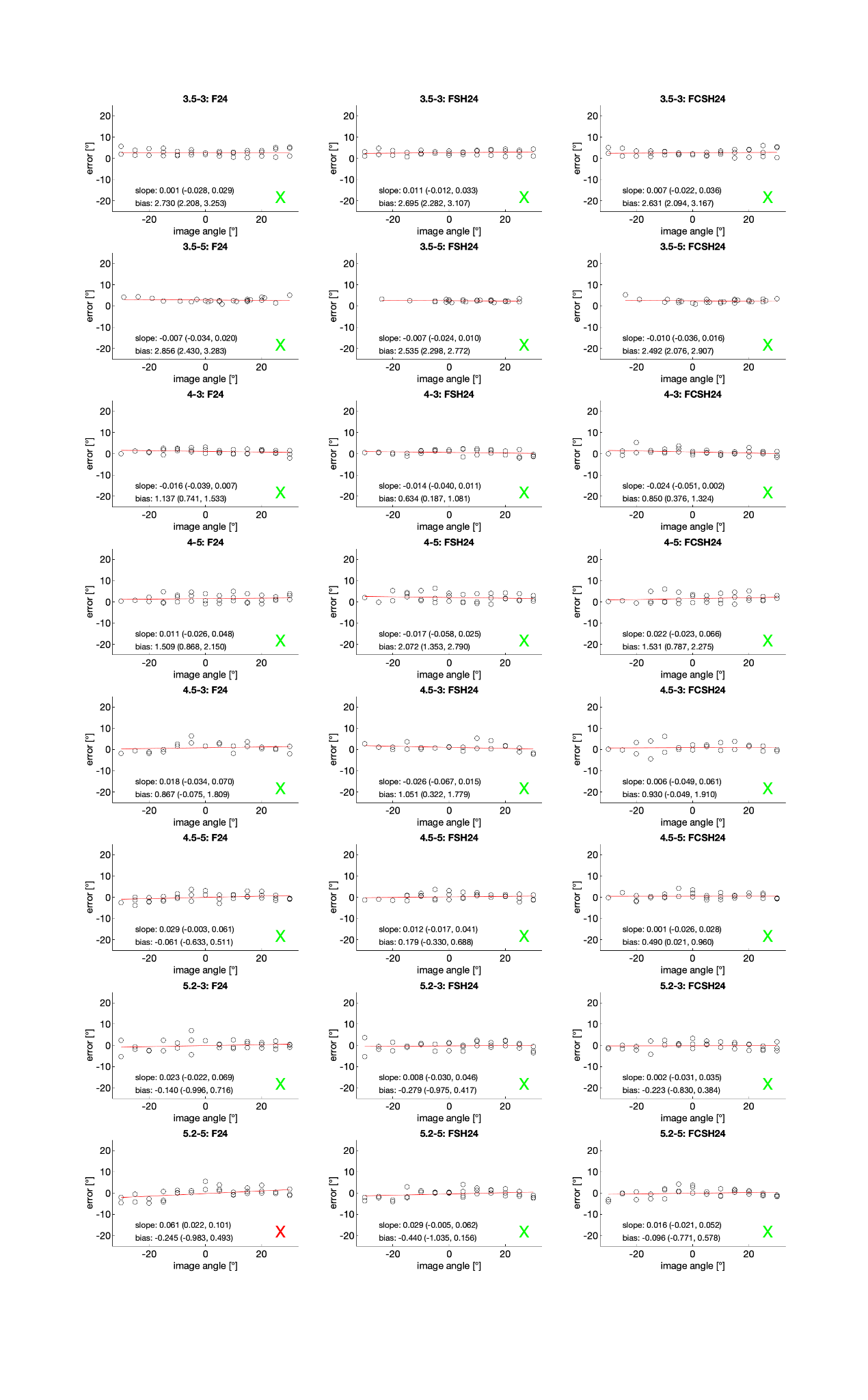 | 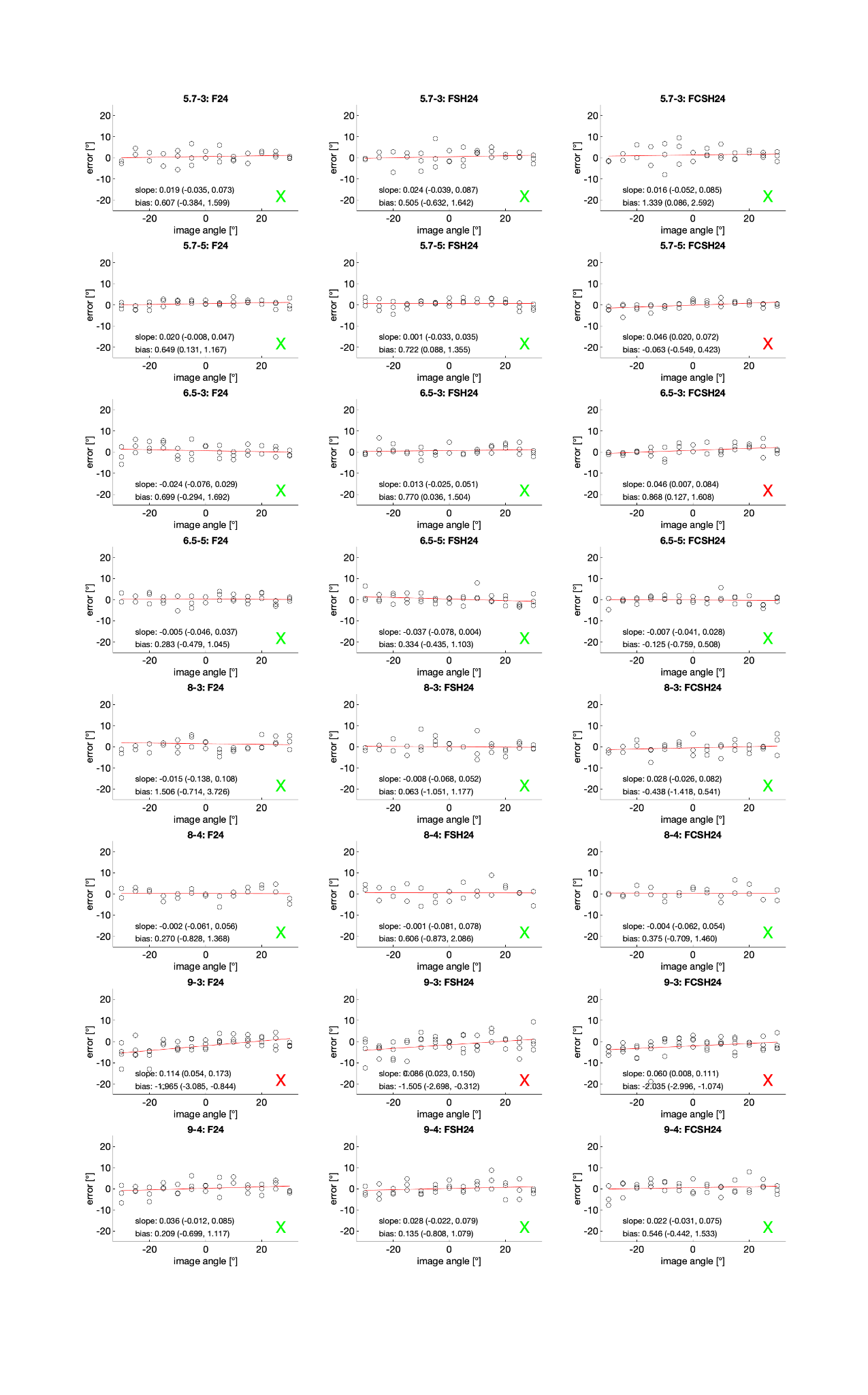 |
| --- | --- |
| **Supplemental Figure S3.** Overview of all abutment angle measurements for all 16 abutment types listed in Supplemental Table S1. Here, analogous to Figure 5, each plot figure also provided information on slope, bias, and image tilt angle dependence. Samples were grouped in sections of 3, corresponding to the 3 imaging modes. There were 8 rows and 2 columns of graphs. Abutment samples 1 to 8 from Supplemental Table S1 were in the first column and the remaining samples in column two. To obtain a general understanding of the data shown in Supplemental Figure S3, statistical summary graphs were generated, shown here in Figures 6, 7 and 8. See Figure 5 for a detailed description of a single case. See Figures 6, 7, and 8 for statistical analysis of these measurements. **Note:** F24, -15° is an example for an erroneous reading due to limited field of view. | |
