# Supplementary material for: Ultrasound insonation angle and scanning imaging modes for imaging dental implant structures: A benchtop study
Source: PLoS One. 2022 Nov 29;17(11):e0270392. doi: 10.1371/journal.pone.0270392 (PMC9707752; doi:10.1371/journal.pone.0270392)
Supplement: S4 Fig — Absolute error (μm) is given as a function of image angle between the implant and the ultrasonic imaging array. Optical measurements (see S2 Fig) serve as a reference standard. Four implant types, i.e., 4.5–13, 4–13, 3.5–13 and 5–13 (left to right) were assessed using 3 imaging modes, i.e., F24, FSH24, and FCSH24 (top to bottom). For each combination of implant and imaging mode the image angle was arranged from -30° to +30° and three estimates of the implant thread pitch were obtained. The difference between the ultrasonic and the optical measurement is plotted as the function of image angle. Finally, the resulting data is fitted using a 1st order polynomial. Slope and bias are provided for each imaging mode. Slope describes dependence of the error with respect to the image angle. Bias describes any constant over- or underestimation of the measurement. For both, the 95% confidence interval is provided in parentheses. A green ‘X’ indicates a slope estimate for which the 95% confidence interval includes zero, i.e., the slope is not significantly different from zero. (DOCX) [file pone.0270392.s004.docx]

| 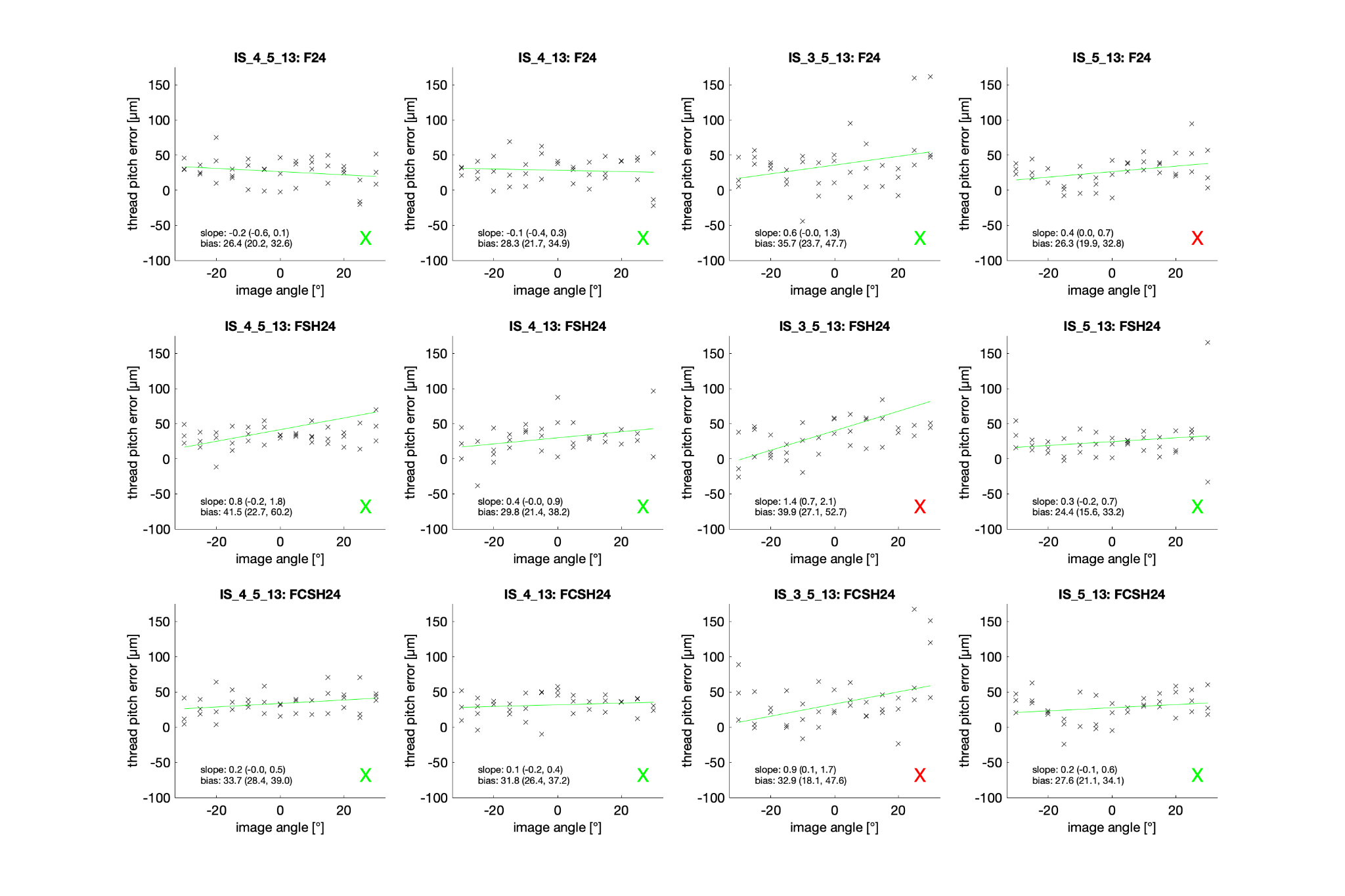 |
| --- |
| **Supplemental Figure S4.** Error in measuring implant thread pitch using high frequency ultrasonic imaging. Absolute error (µm) is given as a function of image angle between the implant and the ultrasonic imaging array. Optical measurements (see Supplemental Figure S2) serve as a reference standard. Four implant types, i.e., 4.5-13, 4-13, 3.5-13 and 5-13 (left to right) were assessed using 3 imaging modes, i.e., F24, FSH24, and FCSH24 (top to bottom). For each combination of implant and imaging mode the image angle was arranged from -30° to +30° and three estimates of the implant thread pitch were obtained. The difference between the ultrasonic and the optical measurement is plotted as the function of image angle. Finally, the resulting data is fitted using a 1^st^ order polynomial. Slope and bias are provided for each imaging mode. Slope describes dependence of the error with respect to the image angle. Bias describes any constant over- or underestimation of the measurement. For both, the 95% confidence interval is provided in parentheses. A green ‘X’ indicates a slope estimate for which the 95% confidence interval includes zero, i.e., the slope is not significantly different from zero. |
