# Supplementary material for: Ultrasound insonation angle and scanning imaging modes for imaging dental implant structures: A benchtop study
Source: PLoS One. 2022 Nov 29;17(11):e0270392. doi: 10.1371/journal.pone.0270392 (PMC9707752; doi:10.1371/journal.pone.0270392)
Supplement: S1 Table — A total of 16 abutments were tested. Note: Abutments are labeled using the smaller angle, i.e., either the opening angle or its complement. Here, for abutments 3.5–3, 3.5–5, and 4–3, the complement angles are listed. (DOCX) [file pone.0270392.s006.docx]

| **S1 Table.** Listing of optical abutment angle reference measurements (N=3 per abutment). A total of 16 abutments were tested. Note: Abutments are labeled using the smaller angle, i.e., either the opening angle or its complement. Here, for abutments 3.5-3, 3.5-5, and 4-3, the complement angles are listed. | | | | | | | |
| --- | --- | --- | --- | --- | --- | --- | --- |
| Abutment number | Abutment  name | No. of scan attempts | Optical measurement (degrees) | | | Mean (degrees) | Coefficient of variation |
|  |  |  | No. 1 | No. 2 | No. 3 |  |  |
| 1. | 3 | 3 | 1.22 | 1.15 | 1.44 | 1.3 | 11.9% |
| 2. | 3.5-5 | 3 | 0.96 | 0.52 | 0.678 | 0.7 | 31.0% |
| 3. | 4-3 | 3 | 7.46 | 7.34 | 7.35 | 7.4 | 0.9% |
| 4. | 4-5 | 3 | 4.91 | 4.72 | 4.57 | 4.7 | 3.6% |
| 5. | 4.5-3 | 3 | 12.34 | 12.39 | 12.25 | 12.3 | 0.6% |
| 6. | 4.5-5 | 3 | 8.33 | 8.34 | 7.98 | 8.2 | 2.5% |
| 7. | 5.2-3 | 3 | 19.99 | 19.79 | 20.35 | 20.0 | 1.4% |
| 8. | 5.2-5 | 3 | 12.72 | 12.39 | 12.3 | 12.5 | 1.8% |
| 9. | 5.7-3 | 3 | 24.38 | 24.12 | 23.97 | 24.2 | 0.9% |
| 10. | 5.7-5 | 3 | 14.75 | 14.64 | 14.45 | 14.6 | 1.0% |
| 11. | 6.5-3 | 3 | 30.39 | 30.36 | 30.09 | 30.3 | 0.5% |
| 12. | 6.5-5 | 3 | 19.31 | 19.39 | 19.21 | 19.3 | 0.5% |
| 13. | 8-3 | 3 | 40.33 | 40.31 | 40.02 | 40.2 | 0.4% |
| 14. | 8-4 | 3 | 32.72 | 32.42 | 32.38 | 32.5 | 0.6% |
| 15. | 9-3 | 3 | 48.1 | 47.81 | 47.51 | 47.8 | 0.6% |
| 16. | 9-4 | 3 | 37.57 | 37.66 | 37.63 | 37.6 | 0.1% |
