# Supplementary material for: Ultrasound insonation angle and scanning imaging modes for imaging dental implant structures: A benchtop study
Source: PLoS One. 2022 Nov 29;17(11):e0270392. doi: 10.1371/journal.pone.0270392 (PMC9707752; doi:10.1371/journal.pone.0270392)
Supplement: S2 Table — True thread pitch (last column) was obtained from the manufacturer specifications. (DOCX) [file pone.0270392.s007.docx]

| **S2 Table.** Listing of optical implant reference thread depth measurements (5 estimates per implant, with 3 repetitions each). True thread pitch (last column) was obtained from the manufacturer specifications. | | | | | | | | | | |
| --- | --- | --- | --- | --- | --- | --- | --- | --- | --- | --- |
| No. | Implant (n=4) | No. scan attempts | Thread depth [mm] (mean of n=4 after one outlier removal) | | | | | Mean | CoV | True pitch  [mm] |
|  |  |  | 1^st^ | 2^nd^ | 3^rd^ | 4^th^ | 5^th^ |  |  |  |
| 1. | 3.5-13 | 5 | 0.24 | 0.36 | 0.36 | 0.34 | 0.33 | 0.348 | 15% | 0.8 |
| 2. | 4.5-13 | 5 | 0.37 | 0.33 | 0.35 | 0.34 | 0.34 | 0.340 | 8.2% | 0.8 |
| 3. | 4-13 | 5 | 0.33 | 0.36 | 0.34 | 0.31 | 0.28 | 0.335 | 21% | 0.8 |
| 4. | 5-13 | 5 | 0.39 | 0.39 | 0.38 | 0.37 | 0.36 | 0.383 | 9.6% | 0.8 |
